# Experiences With mHealth Use Among Patient-Caregiver Dyads With Chronic Heart Failure: Qualitative Study

**DOI:** 10.2196/57115

**Published:** 2024-10-31

**Authors:** Xiaorong Jin, Yimei Zhang, Min Zhou, Xiong Zhang, Qian Mei, Yangjuan Bai, Wei Wei, Fang Ma

**Affiliations:** 1 Department of Nursing The First Affiliated Hospital of Kunming Medical University Kunming City China; 2 Coronary Heart Disease Center Fuwai Yunnan Cardiovascular Hospital Kunming City China; 3 Cardiology Department The First Affiliated Hospital of Kunming Medical University Kunming City China; 4 Digestive Surgery Department The First Affiliated Hospital of Kunming Medical University Kunming City China

**Keywords:** chronic heart failure, informal caregiver, mHealth, experience, dyad

## Abstract

**Background:**

Chronic heart failure has become a serious threat to the health of the global population, and self-management is key to treating chronic heart failure. The emergence of mobile health (mHealth) provides new ideas for the self-management of chronic heart failure in which the informal caregiver plays an important role. Current research has mainly studied the experiences with using mHealth among patients with chronic heart failure from the perspective of individual patients, and there is a lack of research from the dichotomous perspective.

**Objective:**

The aim of this study was to explore the experiences with mHealth use among patients with chronic heart failure and their informal caregivers from a dichotomous perspective.

**Methods:**

This descriptive phenomenological study from a post-positivist perspective used a dyadic interview method, and face-to-face semistructured interviews were conducted with patients with chronic heart failure and their informal caregivers. Data were collected and managed using NVivo 12 software, and data analysis used thematic analysis to identify and interpret participants’ experiences and perspectives. The thematic analysis included familiarizing ourselves with the data, generating initial codes, searching for themes, reviewing themes, defining and naming themes, and producing the report.

**Results:**

A total of 14 dyads of patients with chronic heart failure and their informal caregivers (13 men and 15 women) participated in this study, including 3 couples and 11 parent-child pairs. We constructed 4 key themes and their subthemes related to the experiences with mHealth use: (1) opposing experiences with mHealth as human interaction or trauma (great experience with mHealth use; trauma), (2) supplement instead of replacement (it is useful but better as a reference; offline is unavoidable sometimes), (3) both agreement and disagreement over who should be the adopter of mHealth (achieving consensus regarding who should adopt mHealth; conflict occurs when considering patients as the adopter of mHealth), (4) for better mHealth (applying mHealth with caution; suggestions for improved mHealth).

**Conclusions:**

This study reported that the experiences with mHealth use among patients with chronic heart failure and their informal caregivers were mixed, and it highlighted the human touch of mHealth and the importance of network security. These results featured mHealth as a complement to offline hospitals rather than a replacement. In the context of modern or changing Chinese culture, we encourage patients to use mHealth by themselves and their informal caregivers to provide help when necessary. In addition, we need to use mHealth carefully, and future mHealth designs should focus more on ease of use and be oriented more toward older adults.

## Introduction

Heart failure is defined as signs or symptoms caused by structural or functional cardiac abnormalities with objective evidence of elevated levels of natriuretic peptides and cardiogenic, pulmonary, or systemic congestion [1]. Chronic heart failure (CHF) is a persistent state of heart failure that can be stable, worsening, or decompensated [2]. It is a serious manifestation of the advanced stage of various cardiovascular diseases, is characterized by high morbidity and mortality, and has become more important as a public problem that threatens the lives and health of the world’s population [3]. According to the global heart failure survey, the average incidence rate of CHF is 460/100,000 person-years: 5-year and 10-year survival rates of patients with CHF are only 57% and 35% [4,5], respectively, and about 60% of patients with CHF die within 5 years of diagnosis [6,7]. In addition, a recent multicenter, cross-sectional study suggested that almost one-half of patients with CHF were ≥65 years old [8].

Health Canada defines self-management as “decisions and actions taken by someone who is facing a health problem or issue in order to cope with it and improve his or her health,” and self-management of patients with CHF is defined as the ability to assume responsibility for managing one or more aspects of CHF (eg, symptom monitoring, weight monitoring, medication dosage adjustment, or medication decision-making) [9]. Studies have shown that self-management is significant for patients with CHF; it can effectively improve cardiac function and prognosis and reduce mortality for patients with CHF [9,10]. With the promotion and popularization of smartphones and wearable devices, mobile health (mHealth) has emerged as a new option to support self-management of CHF [11,12]. mHealth is a general term for the use of mobile phones and other wireless technologies in medical practice [13], with common functions including booking of appointments, health information inquiries, and vital sign monitoring. mHealth acts as an extension of the clinical walls and enables a patient-to-provider link regardless of proximity to care, which helps meet the long-term health management needs of patients with CHF, and is widely used in real-time health monitoring, active intervention, and other aspects of care for patients with CHF. Patients with CHF can rapidly contact a provider using mHealth, which can save valuable time in the treatment of such a critical and chronic disease [14,15]. A systematic review showed that mHealth can enhance self-care and medication adherence, increase quality of life, reduce depression and anxiety, improve patient satisfaction, increase cardiac function, and reduce hospitalization rates and hospitalization costs of patients with CHF [15]. These outcomes indicate that mHealth is effective in improving the self-management of patients with CHF, thereby diminishing the burden of care [16,17].

Despite the many benefits of mHealth, there is inconsistent evidence regarding outcomes related to mHealth for managing CHF [14], and there are some problems with mHealth in CHF situations that challenge the practice of mHealth. For example, mHealth, such as remote telemedical management, did not reduce all-cause mortality in ambulatory patients with CHF [18]. In addition, among patients recently hospitalized for CHF, telemonitoring did not improve outcomes [19]. Additionally, some patients have expressed difficulty with downloading electronic health-related equipment and procedures and with practicing mHealth due to the complexity; this increased their anxiety about its use and led to patient dissatisfaction [20-22]. Furthermore, mHealth may not be suitable for all patients with CHF, such as older adult patients with low electronic health literacy. In addition, the limited accessibility of electronic devices, including the fees associated with some services, can affect the practice of mHealth [15]. Hence, patients might not accept mHealth due to these problems, leading to low mHealth use among patients with CHF [23,24]. A systematic review reported that more than 20% of patients with CHF had never used mHealth and 60% of patients with CHF no longer used mHealth after a single experience [25]. In order to increase mHealth adoption among patients with CHF, it is necessary to understand their perceptions of and experiences with mHealth use. Previous studies have stated that patients with CHF are satisfied with the use of mHealth for self-management [26,27]. However, studies have also proposed that patients with CHF experience difficulties using mHealth for self-management and are dissatisfied with it [20-22]. These inconsistent results suggest that this area needs further exploration. Frequent medical appointments are necessary for patients with CHF [28], and when they are unable to use mHealth independently, informal caregivers (ICs) [29] (individuals who provide unpaid care and assistance to friends or family members because of a health condition) play an important role [28,30]. According to the study by El-Dassouki et al [31], IC guidance not only enables patients with CHF to gradually overcome the digital divide but also greatly improves the experience of mHealth use for patients with CHF. Therefore, studies on mHealth experiences only from the perspectives of individual patients with CHF may lead to certain limitations [32,33]. Besides, according to the extension of the Technology Acceptance Model proposed by Davis et al [34], patients might be more likely to use services recommended by people who influence their health decisions, such as ICs [35]. Therefore, the purpose of this study was to explore the experience with mHealth use among CHF patients and their ICs from a dichotomous perspective, which can enrich and improve the data and provide a theoretical basis for future intervention, thereby improving mHealth use.

## Methods

### Study Design and Setting

A descriptive phenomenological study was performed from a post-positivist point of view to explore the dyadic experiences with mHealth use among patients with CHF and their ICs. Descriptive phenomenology advances human understanding by revealing the nature and organized structure of phenomena without imposing preconceived notions that may influence the understanding of the experiences being examined [36]. This approach was chosen due to its emphasis on exploring the essence of the phenomenon as it is lived by the participants, aligning with the study’s focus on understanding the subjective lived experiences of mHealth use among patients with CHF and their ICs [37]. The Standards for Reporting Qualitative Research (SRQR) checklist informed the development, analysis, and reporting of this study [38].

We conducted dyadic interviews, which were defined as semistructured interviews with 2 family members (ie, the patient with CHF and his or her IC) conducted by 1 researcher [39]. Unlike other interview methods, dyadic interviews can obtain the perspectives of patients and ICs at the same time, which helps the researchers to obtain more comprehensive data [40]. Second, dyadic interviews can demonstrate the interaction between the patient and IC, helping the researcher to explore complex perspectives, whether complementary or contradictory [41-43]. In addition, dyadic interviews allow participants to spark ideas that may not have been recognized nor remembered, thus enabling both participants to respond to and build on each other's contributions in their interactions during the interview process [39-41,44].

This study was conducted in the Department of Cardiology at a comprehensive tertiary hospital in Yunnan Province from October 2023 to December 2023. This hospital is located in the provincial capital of Yunnan province and provides services to a wide geographical area of the province and some patients from neighboring provinces. A variety of patients with different backgrounds come to this hospital for its reputation; this patient population might lead to diverse information and rich data about the topic to be studied. A purposive sampling method was used to recruit participants of different ages, genders, occupations, employment status, and types of mHealth resources the patients and ICs were using.

### Participant Recruitment and Selection Process

This study used a purposive sampling method to select patients with CHF who were hospitalized in the Department of Cardiology in a tertiary hospital in Yunnan Province and their ICs from October 2023 to December 2023. The inclusion criteria were (1) 18 years or older for both patients with CHF and ICs, (2) patients were diagnosed with CHF and had at least one IC, (3) at least one participant in a dyad had used mHealth for the management of CHF, and (4) voluntary participation. The exclusion criteria were patients and caregivers (1) with other serious diseases (eg, malignant tumor, severe organ failure), (2) with hearing or speech impairments, or (3) who were also participating in other research projects.

### Data Collection Process

Face-to-face interviews were conducted between the researchers and CHF patient-IC dyad in hospital wards from October 2023 to December 2023. All interviews were conducted by 2 researchers (XJ and YZ), with XJ acting as the primary interviewer and YZ taking field notes. They were current master’s candidate students at a medical school with 1 year to 2 years of experience in a cardiology internship and had received training in qualitative interviewing. The participants and researchers did not know each other, and the interview was conducted only once. With informed consent obtained from the participants, the interviews were audio-recorded. Data collection was based on information saturation, and the absence of new themes emerging determined the number of interviews, resulting in 14 dyadic interviews. The interview outlines mainly included the following: (1) perceptions and experiences of patients with CHF and their ICs concerning mHealth use and (2) expectations of patients with CHF and their ICs about the development of mHealth. The full interview outline is detailed in Multimedia Appendix 1. Interviews lasted between 21 minutes and 42 minutes. Within 24 hours after the interviews, the researcher (XJ) transcribed the audio recordings of the interviews verbatim into textual material. To ensure the accuracy of the content, we asked another researcher (YZ) to re-listen to the recordings and check and verify the transcribed text.

### Data Analysis

Based on Braun and Clarke [45], thematic analysis was carried out to identify and interpret participants’ experiences and perspectives; the thematic analysis included familiarizing ourselves with the data, generating initial codes, searching for themes, reviewing themes, defining and naming themes, and producing the report [46]. We chose thematic analysis for its flexibility and because it allowed us to explore the depth of our data and better understand the commonalities of participants’ discussions and responses. Furthermore, we hoped to generate unanticipated insights using thematic analysis because it allows for social and psychological interpretations of data. Thematic analysis is a family of methods, not a singular method, that includes coding reliability, codebook, reflexive thematic analysis, and thematic coding [47]. We used reflexive thematic analysis in our study, which embraces researcher subjectivity as a resource for research and emphasizes researcher reflexivity. The collated data were imported into the NVivo 12 software [48] for management and analysis. In the initial stages of the analysis, the data were read multiple times to gain insight into its content. Next, the researchers (XJ and FM) each performed independent preliminary coding of the data. After the coding was completed, the researchers compared and discussed the data in order to reach consensus on the final coding. When disagreements arose during the process, YZ intervened to resolve the issue through discussion. After the coding was finalized, we started looking for candidate themes. This process was done by recombining and grouping related codes under the same theme until data saturation was achieved. We defined and named each theme in detail to better understand what each represented. In the final stage of the analysis, the research team met to discuss and refine the existing themes in depth to ensure the accuracy and comprehensiveness of the findings. In the data collection and analysis process, we integrated several strategies to maintain reflexivity. We kept a journal of field notes and a record of the interviewer’s reasoning, judgment, emotional reaction to the interview, and how this influenced the interview. These notes were primarily made after the interviews, and the interviewer recalled as much detail about the interviews as possible. In the data analysis process, we examined previous reflexive journals (field notes and records) from the data collection to provide more context and made additional notes on the transcript to aid understanding [49].

Importantly, all participants were identified by serial numbers (eg P1, P2, where “P” stands for patient, and IC1, IC2, where “IC” stands for informal caregiver). All personally identifiable information was removed from the transcripts. Interview records were stored in a password-secured computer file, and only the identified researcher could access these records. To achieve and ensure the methodological rigor of the study, the 4 criteria of credibility, dependability, transferability, and confirmability were used. To ensure credibility, long-term engagement of the researcher with the data, reflexivity, and peer debriefing were performed. Dependability was ensured by providing a detailed description of the research methods and having peers participate in the analysis process. Transferability was achieved by providing in-depth and rich descriptions of the demographics and geographic boundaries of the study. Confirmability was established using the researchers’ field notes regarding personal feelings, biases, and insights immediately following the individual interviews [50]. Peer debriefing was performed to ensure credibility. Moreover, the disagreements in the research were discussed and resolved through frequent meetings.

### Ethics Approval

The study was approved by the Ethics Committee of the First Affiliated Hospital of Kunming Medical University (2022-L-304). For all the participants, the study was fully explained orally and in written form, and their written informed consent was obtained before the data collection. Participation in the study was entirely voluntary, and no compensation was provided for their involvement. Participant data were deidentified and stored on a local secure server. Further, the participants had the right to withdraw from the study at any time, without giving any reasons. In addition, no identification of individual participants or users in any images occurred in the manuscript or supplementary material.

## Results

### Participants

A total of 14 patients with CHF and their ICs were interviewed in this study, including 3 couples and 11 parent-child dyads. The patients consisted of 9 men and 5 women, with one-half (7/14, 50%) of the patients older than 65 years old, which was similar to the demographics in other research [8]. The ICs were mostly female (10/14, 71%), with an age range of 22 years to 55 years. In terms of the highest level of education, the majority of the patients (9/14, 64%) had primary or junior high school diplomas, while 79% (11/14) of the ICs had obtained a diploma or higher degree. As for the type of mHealth resources the patients and ICs were using, patients used telemedicine platforms more frequently (7/14, 50%), while caregivers were more likely to use mHealth applications (13/14, 93%). In addition, we also collected employment status and economic situations, and the demographic information is detailed in Table 1.

Our qualitative analyses produced 4 salient themes: (1) opposing experiences with mHealth as human interaction or trauma, (2) supplement instead of replacement, (3) both agreement and disagreement over who should be the adopter of mHealth, (4) for better mHealth.

**Table 1:** Demographic characteristics of participants (N=28).

Variables			Patients (n=14), n (%)		Caregivers (n=14), n (%)
**Age (years)**					
	18-45	0		8 (57)	
	46-65	7 (50)		6 (43)	
	66-75	5 (36)		0	
	≥76	2 (14)		0	
**Gender**					
	Male	9 (64)		4 (29)	
	Female	5 (36)		10 (71)	
**Relationship with the patient**					
	Parents	—^a^		0	
	Children	—		11 (79)	
	Couple	—		3 (21)	
**Highest level of education**					
	Primary or junior high school diploma	9 (64)		2 (14)	
	High school diploma	1 (7)		1 (7)	
	College trade or technical diploma	2 (14)		4 (29)	
	Bachelor’s degree or higher	2 (14)		7 (50)	
**Employment status**					
	Retired	4 (29)		1 (7)	
	Unemployed	0		2 (14)	
	Working full-time or part-time	4 (29)		10 (71)	
	Other	6 (43)		1 (7)	
**Monthly income per capita (¥)^b^**					
	<2000	1 (7)		1 (7)	
	2000-4999	4 (29)		4 (29)	
	≥5000	9 (64)		9 (64)	
**Type of mHealth resources the patients and ICs^c^ were using**					
	mHealth application	5 (36)		13 (93)	
	Wearable device	5 (36)		7 (50)	
	Telemedicine platform	7 (50)		11 (79)	

^a^Not applicable.

^b^A currency exchange rate of ¥1=US $0.14 is applicable.

^c^ICs: informal caregivers.

### Theme 1: Opposing Experiences With mHealth as Human Interaction or Trauma

Participants’ experiences with mHealth were polarized, with some saying that using mHealth was a good experience, but others thought that it was an unpleasant experience for them. In this theme, there were 2 subthemes: (1) great experience with mHealth use and (2) trauma.

#### Subtheme 1: Great Experience With mHealth Use

Some patients and ICs reflected that mHealth was convenient because they could get expert suggestions in an easy way. In addition, they considered that online doctors were patient and they answered their questions one by one, no matter how many questions they had, which led to a sense of comfort and humanity compared with their experiences with offline doctors.

Compared with offline appointments, doctors’ attitudes were much better during the online visit, and the responses were quite fast. It feels good.

IC 6

The telemedicine platform sent messages to me periodically, which included health education information and encouragement words, which was a good experience.

IC 4

We are able to book appointments online with leading specialists, find better doctors, and get specialized advice; that is more convenient.

IC 5

#### Subtheme 2: Trauma

However, some participants recalled an experience of being scammed while using mHealth, which was a negative experience for them. These participants stated that, although a long time had passed, they were scared whenever they thought of it, and it was traumatic for them to some extent.

When I surfed online someday, I found a link to a well-known medical expert, which was the one that I wanted to visit but couldn’t because of his busy appointments. Then I clicked the link, filled out the information, and finished the payment with the hope that I could see the doctor in this easy way. I took the payment voucher to the hospital only to know that this was a fake, I was cheated tens of Yuan! That is a terrible experience.

IC 2

### Theme 2: Supplement Instead of Replacement

Most participants stated that, despite the many advantages of mHealth such as convenience and speed that were the result of the digital age, it was constrained by its form and the authenticity and accuracy of the information. They thought that mHealth could not completely replace traditional health-seeking behaviors and it was a supplement to offline hospitals. In this theme, there were 2 subthemes: (1) it is useful but better as a reference and (2) offline is unavoidable sometimes.

#### Subtheme 1: It is Useful but Better as a Reference

In terms of content, patients and ICs suggested that mHealth could provide some information about their illnesses and medications, but they were troubled by the inconsistent advice that emerged from their inquiries. Hence, mHealth was better used as reference in their point of view.

It’s useful to know what’s wrong with your body online, but sometimes I don’t know how to identify the right information, and it might be better to be used as a reference.

P 7

#### Subtheme 2: Offline is Unavoidable Sometimes

All participants reflected that mHealth had some limitations and restrictions. Sometimes, they felt the information that mHealth could provide was vague, which did not really solve their problem. Furthermore, the online delivery of tests and treatments was impossible.

During an online consultation, the doctor gave you a general idea; in the end, you have to go to the hospital for examination and treatment.

IC 4

### Theme 3: Both Agreement and Disagreement Over Who Should be the Adopter of mHealth

In the dyad data, we found that patients’ and ICs’ views on who should adopt mHealth demonstrated both consensus and conflict. In this theme, there were 2 subthemes: (1) achieving consensus regarding who should adopt mHealth and (2) conflict occurs when considering patients as the adopter of mHealth.

#### Subtheme 1: Achieving Consensus About Who Should Adopt mHealth

In this subtheme, patients and ICs agreed on who should use mHealth. For the dyads reaching a consensus, a minority of dyads agreed that patients should use mHealth by themselves. In this situation, patients stated that they used mHealth by themselves, and their ICs agreed with them, considering that patients should have the ability to use mHealth for health care and disease management. However, most dyads who reached consensus agreed that ICs should use mHealth for the patients. The patients reflected that they were not capable of using mHealth independently due to age, illness, and other factors and ICs should do it all, which was also supported by their ICs, who held the belief that patients were incapable of using mHealth and they should use mHealth for their patients.

I can make an appointment to see the doctor and buy medicines on the mobile phone...I can solve the problems by myself, so I don’t trouble my children. I suppose that can reduce the burden on my children.

P 5

I support my dad with using mHealth for registration and health information; he has no problem with it. I am not always able to be with my father, and I hope that he has the ability to use mHealth.

IC 5

I’m old. I can’t see well, I can’t read much, I can’t type, and it’s too difficult to use (mHealth).

*P* 14

My father is getting older...he has heart failure, and I will help him as much as I can. It may be impractical for him to use it (mHealth) on his own; after all, he is too old.

IC 14

#### Subtheme 2: Conflict Occurs When Considering Patients as the Adopter of mHealth

Within this subtheme, there was conflict between patients and ICs regarding users of mHealth. Some patients thought of themselves as the adopter of mHealth because they believed in their ability to do it, whereas their ICs didn’t support patients’ use of mHealth, as they held the belief that patients were vulnerable and could be easily cheated or it was hard for patients to express themselves online. In addition, a few patients reflected their unwillingness to use mHealth because of their vulnerability, yet their ICs insisted that the patients should do their jobs.

I think these things (mHealth) are simple, no big deal, I can master them myself, but my daughter won’t let me use them myself...It’s my disease. I want to manage it myself.

P 9

I will not recommend my father to use mHealth; there are some risks. For example, the elderly are likely to consult Baidu (an encyclopedia website in China), which is one-sided and often scares you. I don’t want my father to search Baidu; the information from Baidu is not completely correct. To make things worse, the information from Baidu adds a heavy psychological burden to my father.

IC 9

I can’t; I already suffer from heart failure, my memory is failing, I’m little educated, and I don’t think about these things (mHealth), I just think about living a few more years, and that’s just fine.

P 2

We all have our things now, that is, it is unrealistic to accompany the elderly for a long period of time. He (my father) should manage his condition, including using mHealth.

IC 2

### Theme 4: For Better mHealth

Participants reflected that they had applied some skills in using mHealth, which could be more convenient. In addition, they also made some suggestions for the future development of mHealth. In this theme, 2 subthemes were generated: (1) applying mHealth with caution and (2) suggestions for improved mHealth.

#### Subtheme 1: Applying mHealth With Caution

Patients and ICs mentioned that they could ensure the accuracy and authenticity of the information by using official channels, multiple queries, and double-checking strategies when using mHealth.

I'll read much more information about my question on the internet, and finding the official website is necessary. Anyway, I would not trust the information if it had a contradiction. If possible, I will confirm with some experts.

IC 1

#### Subtheme 2: Suggestions for Improved mHealth

Patients and ICs offered several suggestions in terms of improving the mHealth experience, including stricter regulations, more official channels, simpler steps, and fee deductions for online consultation services.

For these online environments, published content should be more strictly regulated.

*P* 10

It is best to have a way to allow me to identify the true and false, such as official channels.

P 7

The (mHealth) easier the better, with no barriers is the best. I wish my father could learn to operate it for its simplicity.

IC 2

The expert fee (Online Clinic Service) is too expensive. I asked her four or five questions for five or six hundred dollars, and this price is too high. If it can be cheaper, it would be better.

IC 10

## Discussion

### Principal Findings

The results of current reports on mHealth experiences are mixed [13,51], which is consistent with our findings. Our findings are supported by those by Agnihothri et al [13] and Hilty et al [52]: mHealth can provide convenient care to patients and improve the efficiency of medical visits. However, some studies have shown that online medical visits lack the essential element of a doctor-patient relationship, which constitutes the human touch [53,54]. Our findings suggest that mHealth can provide users with a more comfortable health care experience and has characteristics of humanity as well, which is a novel finding. This might be explained by the theory of bureaucratic caring [55,56], which suggests that, under pressure to perform work with maximal efficiency in a minimal amount of time, doctors might pay more attention to enhancing the care of technological dimensions such as disease diagnosis and treatment, especially in the midst of numerous patients and high patient demands [57]. In contrast, online doctors have more time and less pressure due to the virtual environment of the work, which highlights the advantage of mHealth. In addition to the aforementioned advantages, we identified some concerns with mHealth. Previous studies have suggested that mHealth suffers from a lack of standardized diagnostic criteria and unregulated and invalidated processes or technologies, which significantly increase the risk of telecommunication fraud [13]. In this study, participants described their experiences of being scammed, and these ultimately led to an unpleasant experience, which highlights the importance of network security.

Previous studies have noted that, during the process of searching for health information, users usually retrieve a large amount of information of varying quality [58], which makes it difficult for users to discern the authenticity of the information, and this is why the results of this study support the idea that mHealth information is just for reference. Not only that, the limitations of the mHealth format lead users to obtain incomplete data through online counseling [59], and it is challenging to perform physical examinations and treatments online [60-62], making it difficult for doctors to make the correct diagnosis, which ultimately leads to incorrect referrals and poor outcomes [54]. The aforementioned reasons have led to patients trusting face-to-face medical consultations more, especially patients with CHF who often experience a life-threatening deterioration of their condition. Patients with CHF need medication or surgical treatments that mHealth cannot provide, making it necessary for them to go to the hospital to solve their problems.

In our study, we attempted to highlight the added value of using the dyad as the unit of analysis while collecting data from individual participants in qualitative research. While contrasting and overlapping 2 individual versions, we captured a third dyadic relationship (created by the researchers) without losing or corrupting the individual versions, and the dyadic perspective added dimension to our understanding [39]. First, using overlapping, we constructed a subtheme that patients with CHF and their ICs reached an agreement as to who should be the adopter of mHealth. The consensus was reached in 2 contexts: One was that patients and their ICs considered that patients should use mHealth, and the other was that patients and their ICs agreed that it was the ICs’ responsibility to use mHealth for patients. The use of mHealth by patients highlights guaranteed patient empowerment, which is viewed as a key factor for improving health outcomes and bringing about better adherence with treatment regimens [63]. In addition, when patients are empowered, they are more likely to take an active role in medical encounters, have a better understanding of different treatment options, and participate more in shared decision-making [64]. Furthermore, in Chinese culture, Confucius dictated that the senior partner owes the strong duties of care and benevolence to the junior partner. To avoid troubling their children in the parent-child pairs, parents might choose to use mHealth by themselves. In other cultural backgrounds, older patients with heart failure had high confidence for using telemonitoring devices, demonstrating that older adult patients with heart failure are ready to embrace mHealth management programs [65]. However, our study also suggested that it was the caregivers’ responsibility to use mHealth, which is common in the Chinese cultural context. The Chinese are heavily influenced by Confucianism, in which Five Cardinal Relationships exist, requiring that, in the parent-child dyad, the junior partner (child) owes a strong duty of service and reverence to the senior partner (parent). As in this study, ICs in the parent-child pairs, instead of the patients, used mHealth to help them manage their illnesses and, in this way, showed their caring for family members. Similarly, in the parent-child dyad, patients also considered the responsibility of using mHealth should be put on their caregivers to show respect for their parents and loyalty to the family. In contrast, western countries have more robust social security and welfare systems that prioritize social pensions; hence, the caregivers’ responsibilities are less pronounced in western societies [66].

Second, by studying the contrasts between the 2 individual versions, conflicts occurred. Studies have pointed out that a high prevalence of dyadic incongruence occurs in heart failure dyads, with disagreements on illness management that were not resolved, leading to poorer adherence to medical recommendations, worsening CHF symptoms, and damage to the dyadic health [67,68]. In our study, the incongruence was demonstrated as either the patients considered themselves as the adopter of mHealth but their ICs disagreed with the patients’ independent use or the patients were unwilling to use mHealth but their ICs wanted the patients to use it. The former is associated with ICs’ concerns about the potential risks of mHealth use by the patient. Previous studies have noted negative stereotypes of older adults as unmotivated, sickly, stubborn, and gullible [69,70]. Hence, caregivers, especially children in the parent-child dyads, refused to allow patients to use mHealth because they believed that patients would be deceived or make mistakes in the process. However, patients wanted to be the first person to be responsible for their health, which led to the conflict. The latter may be related to the Chinese culture becoming more individualistic [71]. Previous research has shown that, in individualistic cultures, family structures tend to be freer and looser than in collectivistic cultures, which leads to people living separately from other family members as well as smaller families [71,72]. In this study, there were instances where ICs and patients did not live together, and they expected patients to be able to use mHealth on their own when experiencing illness-related problems. This might be explained by the fact that the children in the parent-child dyads might hold the individualism aspect and prefer equity; they are much less concerned with relationships and take a flexible view toward social obligations, which might be due to the fact that filial piety and family obligations have to compete for resources with the demands of a modern society and the needs for personal achievement of family members [73]. However, older adult patients may refuse to use mHealth due to sensory impairment, cognitive decline, and memory loss [31], which leads to conflict as to who should use mHealth. When managing a chronic disease, people are increasingly seen as coproducers of their health and need to be empowered to take control of the determinants of their health [63]. According to systematic reviews, self-management support interventions are associated with improved outcomes among people with comorbid diabetes and chronic kidney disease, and self-management improves type 2 diabetes treatment by helping people stay healthy and adapt to their illness, highlighting the key role of self-management in chronic disease management [74,75]. Therefore, patients should be encouraged to use mHealth by themselves, and their ICs should help them if needed. Furthermore, with the juxtaposition of tradition and modernization and globalization, one might expect to see gradual shifts from interdependence to independence in Chinese culture, particularly in the younger generations [73]. Hence, self-reliance is advocated, especially in the management of chronic disease, which emphasizes that patients should use mHealth, with or without the support of their caregivers.

To achieve better effectiveness of mHealth, participants summarized several tips, including repeated queries, multiple comparisons, and the use of official channels. This is consistent with the study by Jia et al [76], which showed that more than 60% of health information consumers searched at least 3 different websites to locate and track health information and that authoritative and official channels were more trusted by consumers. We suggest that future mHealth designs should focus more on ease of use and user-friendliness for older adults, which is also supported by the findings of Zhao et al [58]. Consistent with the findings of Dang et al [77], we would like to see lower fees for mHealth services in the future. In addition, participants would like to have stricter regulations and auditing to help them access more accurate health care resources.

### Limitations

This qualitative study has obvious strengths but also limitations. It compensates for the lack of existing data on the experiences with mHealth use by conducting dyadic interviews with patients with CHF and their ICs. However, only patients and 1 of their ICs were interviewed, and other family members’ perspectives were ignored. This study was conducted in the context of CHF, so the results may not be easily generalizable to other illnesses. Future studies could be conducted more deeply on a family basis and in different diseases. In addition, this study involved only a small group of people with CHF and their caregivers in 1 hospital of Yunnan Province, China, which reduces the generalizability of this study, and caution should be taken when reading the results.

### Conclusions

Our study reported that experiences with using mHealth varied between patients with CHF and their ICs. The fact that mHealth involves the human touch is emphasized in our study. Although we acknowledge the convenience and efficiency of mHealth, we also suggest that users should be aware of network security while using it. In addition, mHealth should complement rather than replace offline hospitals, which are more practical for solving problems. Meanwhile, in the context of the modern or changing Chinese culture, we highlight that patients should use mHealth and ICs should only provide help and guidance when necessary. In addition, our study proposes that mHealth use should be cautious, and the design of mHealth should be more tightly regulated, more affordable, simpler, and more friendly to meet the needs of older adults in the future.

## Appendix

Multimedia Appendix 1 Interview guide.

## Abbreviations

CHF: chronic heart failure
IC: informal caregiver
mHealth: mobile health
SRQR: Standards for Reporting Qualitative Research

## Data Availability

The full interview transcripts and audio files are not available to the public due to data protection reasons. However, transcripts and findings are available on request from the corresponding author.
