# Supplementary material for: Experiences With mHealth Use Among Patient-Caregiver Dyads With Chronic Heart Failure: Qualitative Study
Source: J Med Internet Res. 2024 Oct 31;26:e57115. doi: 10.2196/57115 (PMC11565088; doi:10.2196/57115)
Supplement: Multimedia Appendix 1 [file jmir_v26i1e57115_app1.docx]

**Interview guide**

1. Have you used mHealth?

2. Did you have any impressive experience in using mHealth? Can you describe them specifically? (used); what hindered you from using mHealth (not used).

3. Please talk about your views on mHealth? (Strengths, weaknesses and how it compares to offline hospitals).

4. How do you manage chronic heart failure using mHealth?

5. What are your suggestions and expectations for mHealth?

6. Do you have any other experiences or feelings about mHealth that you would like to express?
